# mHealth based intervention by social care professionals to support family caregivers to persons with dementia living at home in Sweden (Caregiver Connect): a randomized controlled trial

**DOI:** 10.1186/s12877-024-05106-x

**Published:** 2024-06-14

**Authors:** Zarina Nahar Kabir, Marie Tyrrell, Hanne Konradsen, Åsa Craftman, Nitin Joshi, Manoj Kumar Gupta, Suresh Sharma, Pankaj Bhardwaj

**Affiliations:** 1 Division of Nursing, Department of Neurobiology, Care Sciences and Society, Karolinska Institutet, Alfred Nobels Allé 23, Huddinge, 141 52 Sweden; 2 Department of Nursing, Sophiahemmet University, Valhallavägen 91, Stockholm, 11428 Sweden; 3 School of Public Health, Department of Community Medicine and Family Medicine, All India Institute of Medical Sciences, Jodhpur, 342005 India; 4 College of Nursing, All India Institute of Medical Sciences (AIIMS), Jodhpur, 342005 India

**Keywords:** Dementia, Dementia care, Family caregiving, Family member, Family research, Family support, Immigrant, mHealth, Neurocognitive disorder

## Abstract

**Background:**

The majority of persons with dementia in Sweden reside in their own homes with support from family members. Approximately, 12% of persons with dementia have immigrant background. Within the next 20 years, the number of persons with dementia who are non-ethnic Swedes is said to double. Family caregivers with immigrant backgrounds are noted to receive less support in the community than ethnic Swedes and rate their health status lower than ethnic Swedish peers. The Swedish National Board of Health and Welfare have highlighted the importance of follow-up support for family caregivers with immigrant backgrounds as there is a recognized gap in research and available information tailored to meet the needs of this group.

**Purpose of the study:**

The purpose of the study is to test effectiveness of an mHealth based intervention through which community social workers can improve caregiving competence of non-European immigrant family caregivers of people with dementia living at home in Sweden. The overarching aim is to reduce caregiver burden and depressive symptoms, and improve quality of life.

**Methods:**

A randomized controlled trial (RCT) including wait list control group will be performed consisting of an intervention group (A, *n* = 44) and a wait list control group (B, *n* = 44), totaling a sample size of 88. On completion of the 10-weeks long intervention in the intervention group, the intervention will be delivered to group B. Effect of the intervention will be analyzed between and within groups over time. The content of the educational component of the intervention is inspired by the iSupport manual developed by the World Health Organization. The contents, in the form of a booklet, aims to equip the family caregivers with structured information on understanding dementia as a condition and its management at home, including self-care guidance designed specifically for family caregivers themselves.

**Discussion:**

Similar telephone-delivered intervention studies targeted for family caregivers to persons with dementia are ongoing in Malaysia and will start in India using the same booklet adapted to the local context. These studies will provide evidence on the effectiveness of using digital technologies to deliver support to those who may not be reached or adequately served by the traditional healthcare system.

**Trial registration:**

ISRCTN registry, Registration number ISRCTN64235563.

**Supplementary Information:**

The online version contains supplementary material available at 10.1186/s12877-024-05106-x.

## Background

In Sweden, it is estimated that one in every four persons will be 65 years or above by the year 2040 [1]. As a result of an increased aging population with persons aged 80 years and over, the prevalence of dementia is expected to rise from approximately 150,000 in 2012 to 250,000 people by the year 2050. Most persons with dementia, between 55% and 72%, reside in their own homes [2, 3] with support from family members [4]. In 2018, the number of people living with cognitive diseases (dementia) with immigrant backgrounds in Sweden was estimated at 20,000 and set to double within the next 20 years [5]. According to the national Swedish Dementia Register, 12.4% of the registered persons with dementia have immigrant background [6].

Family caregiving plays a pivotal role in all long-term care systems, this is apparent in areas where formal long-term care services are lacking or absent. It is deemed a cost-efficient way of delaying admissions to residential facilities in high-income countries and facilitates the person receiving care to reside in their own home. During the Covid-19 pandemic, the role of family caregivers became more evident in Sweden [7]. According to the European Commission, an informal caregiver is described as a person who cares for or provides assistance to a person with disabilities without financial reimbursement or formal education in health care [8]. It is often family members who assume the role of informal caregivers and are the main source of support for older persons with complex care requirements [9]. In Sweden, one in five adults provide care for an older family member where informal care is regarded as the main source of care provided [7].

In a study of over 170 family caregivers to persons with dementia in Cyprus, 68% experienced elevated levels of burden (more women than men), with 65% reporting symptoms of depression [10]. In the early stages of the dementia disease trajectory, family caregivers are often not identified as needing support by health and social care professionals despite many experiencing depression, anxiety, and insomnia [11]. The level of care provided can often be beyond the caregiver´s capacity and can result in experiences of burden and mental health disorders [12]. Caregiver competence in caring for older frail persons is related to depression among caregivers. Possibilities exist for supporting caregivers by providing targeted interventions which can enhance their competency and mastery in the field [13]. The impact of caregiving on family life, finances and mental health are key components of family caregiver burden [14]. Family caregiving is diverse and complex by nature with roles evolving over time as the disease progresses with caregivers often unprepared for the role [15].

Almost 20% of the population of Sweden (two million people) have an immigrant background. There has been a rise in immigration to Sweden by 9% over the past ten years. The majority of people come from the Middle East (Syria and Iraq) followed by Finland, Poland, and Iran [16]. There is inequity in care provided to persons with dementia with immigrant background compared to ethnic Swedes in regard to clinical assessment, treatment and health care [17]. Family caregivers with immigrant backgrounds are noted to receive less support in the community in the form of home help, respite care and information than ethnic Swedes. Family caregivers with immigrant background have rated themselves as having lower levels of health than their peers and are living with concerns and challenges in relation their situations [18]. In Sweden in 2012, 1.3 million informal caregivers with one in six aged 60 years and older identified themselves as providing care for their cohabitating partner [19]. The response to this survey among people born outside of Sweden was low. The Swedish National Board of Health and Welfare have highlighted the importance of follow up support for family caregivers with immigrant backgrounds as results of previous studies do not represent this group.

The World Health Organization highlights, in their guidelines for community-based interventions for integrated care for older people, the need to provide support to family and informal caregivers who care for older people [20]. Areas of importance for caregivers are emotional support, help accessing and navigating healthcare systems, support with daily living and managing the situation. In Sweden, research shows the importance of advocating the value of a person-centered approach to meet the specific needs of family caregivers to persons with dementia. This is especially relevant for family caregivers who experience a lack of support in managing behavioral and cognitive symptoms in caring for their family member with dementia. Family caregivers can feel vulnerable and socially isolated in their caring role [21]. According to the Ministry of Health and Social Welfare, the Social Services Act (2001:453) in Sweden states that the municipalities in the country are responsible for providing support services for family caregivers who care for persons with chronic illnesses, persons with function variations and older persons [22]. In Sweden, health and social care workers, e.g. dementia care nurses and social workers supporting family members, work both at regional levels and locally in the municipalities to provide support for families living with dementia. Support offered includes providing information and guidance. Despite this a Swedish study showed that 75% of family caregivers were not aware of the availability of such support [23]. When family caregivers´ needs and wishes for information are unmet [24], availability of information via technology can be one way to strengthen their role and help them make informed decisions.

Mobile health, otherwise known as mHealth, is a form of telehealth and in general terms describes the use of mobile and wireless technology in health care [25]. Mobile phones or tablets are commonly used [26]. The goal of mHealth is to promote delivery, health outcomes and efficiency in health care systems [26]. In a literature review by Zhao et al. [27], factors which contributed to care efficiency while using mHealth included that the applications were user friendly, provided personalized support and allowed accessibility in communicating with health care professionals. Over the last two decades, the availability of interventions to reduce family caregiver burden within health care has increased, however such interventions have mainly been carried out face to face. A rise in the use of information and communication technology (ICT) has opened up for novel possibilities to support family caregivers where geographical distances from services or lack of resources in the community have been viewed as barriers to support. An example of an ICT based intervention supporting family caregivers to persons with dementia is Diapason, a web-based automated educational program [28]. Recent research promotes the need for further development of smartphone based mobile applications focusing on the complex needs of family caregivers in providing support for a person with dementia [29].

## Methods

### Aim

The aim of the randomized controlled trial described in this study protocol is to test effectiveness of an mHealth based intervention through which community social workers can improve caregiving competence of non-European immigrant family caregivers of people with dementia living at home in Sweden. The overarching aim is to reduce caregiver burden and depressive symptoms, and improve quality of life.

### Research questions

- Is mHealth-based intervention delivered by community social workers effective in reducing caregiver burden and depressive symptoms of non-European immigrant family caregivers to people with dementia living at home?
- Is mHealth-based intervention delivered by community social workers effective in improving quality of life of non-European immigrant family caregivers to people with dementia living at home?

### Study design

This randomized controlled trial (RCT) will include a wait list control group. Participants will be randomly assigned to the intervention group and the wait list control group. On completion of the 10-weeks long intervention in the intervention group (Group A), the intervention will be delivered to the wait list control group (Group B). Effect of the intervention will be analyzed between and within groups over time.

### Definitions

Family caregiver of Person with Dementia (PWD) is defined as one who provides informal care, hereupon referred to as ***family caregivers***.

### Community workers

The term community workers indicate Social Care Professionals (SCP) based in municipalities in Sweden. They include professionals, i.e., social workers, Silvia Nurses (specially trained in dementia care) and dementia coordinators providing support to family caregivers of people with dementia. SCPs in the selected municipalities will select family caregivers through their own network in their catchment area.

### Study sites

The research will be conducted in partnership with SCPs in municipalities with high immigrant density in Stockholm County (and other counties if the required sample size is not reached) in Sweden.

### Study participants and inclusion criteria

Participants in the RCT will include (i) family caregivers of PWD living at home. The family caregivers are the recipients of the intervention. In the case of more than one family caregiver for a PWD, they will be included either in the same intervention or the same wait list control group; and (ii) SCPs who will deliver the intervention. Inclusion criteria of family caregivers are adults of non-European immigrant background in Sweden, who have provided care to a PWD living at home for at least six months, possess a smartphone or tablet, has access to the internet at his/her own cost, and able to read and write Swedish. Family caregivers aged under 18 years, and/or those suffering from conditions that impede communication will not be included in the study.

### Recruitment of participants

#### Quantitative component

Sample size calculation of family caregivers is based on the primary outcome caregiver burden assessed by Zarit Burden Interview [30]. Assuming an effect size of 0.70, alpha 0.05, power 0.80, and 25% refusal or drop-out rate, the required sample size will be 44 family caregivers each in intervention group and wait list control group, totaling a sample size of 88. Recruitment of family caregivers will be done in multiple ways. The SCPs of the municipalities selected for the study will announce to all in the network of the family caregivers of PWD, as defined above, who are in their catchment area. Announcement about the project and invitation to join the study will be published in the local newspaper if necessary. Cultural and community organizations of different ethnic minority groups will also be reached out with information on the project. Participants will be randomized 1:1. To ensure allocation concealment, a sealed opaque envelope containing 88 small pieces of paper with numbers 1–88 written on it. Of these, 44 will be marked with “I” to indicate intervention group and another 44 with “C” to indicate control group. Once a participant is enrolled in the study, a person outside the research group will randomly pick a piece of paper from the envelope to indicate if the enrolled participant will belong to the intervention group or the wait list control group.

After inclusion, the SCP will contact a designated member of the research team who will draw a sealed envelope which will decide the allocation to either control or intervention group. A SCP selected for the study will be responsible for 5–7 family caregivers at a time for delivering the intervention. Those family caregivers not selected in the initial intervention group, i.e., those in the wait list control group, will continue to receive the standard support. Recruitment of study participants is expected to begin in August 2024.

#### Qualitative component

After completion of the intervention, 15–20 family caregivers who have received the intervention will be purposively selected for in-depth interviews. All the SCPs delivering the intervention will also be interviewed about their experience of delivering the intervention.

### The intervention

This study will assess the effectiveness of an intervention delivered via a mobile application by SCPs in reducing caregiving burden and depressive symptoms, and improving quality of life of family caregivers of persons with dementia by improving their caregiving competence.

#### Educational component of the intervention

The educational component of the intervention will be delivered by SCPs in Sweden via a mobile app. The intervention will be delivered in 10 sessions with specific topics for each session and which is based on World Health Organization’s recommendation of support for family caregivers. The topics include an introduction to dementia, being a caregiver, mental strategies and mind-set, activities, diet and sleep, and where to find resources in the community. The family caregiver will receive a booklet, presenting each topic and a minor task which will be followed up in the next session with the SCP. One of the 10 sessions will be kept as an open choice allowing the family caregiver to freely address any topic pertinent to their unique situation. The content of the intervention will be refined based on consultative workshops with stakeholders including SCPs, immigrant family caregivers of PWD, older persons of immigrant background and experts.

In addition to SCPs delivering the intervention through the chat feature on a mobile application, other features of the app will include:

1. ***Interaction with community workers***: Using the chat feature, family caregivers will be able to communicate directly with the SCPs on a one-on-one basis throughout the 10-week intervention period.
2. ***Relevant services***: Collection of weblinks of relevant services in their communities will be available.
3. ***Peer support***: The family caregivers will be able to chat with their peers, i.e., other family caregivers of persons with dementia, at the time of their preference.
4. ***Wellbeing***: Mindfulness exercises will be available for the family caregivers.
5. ***Diary***: The family caregivers will be able to write down personal notes and reflections.

#### Training of SCPs

All SCPs will receive a short training introducing them to the mobile app and familiarizing them with contents of the intervention. The training will be provided by a team of two nurses specialized in dementia care and care of older persons with expertise in training nurses in dementia care at university level education.

The research team and the SCPs will meet virtually at least twice during every cycle of the 10-week long intervention period to discuss and calibrate their approach in their interaction with family caregivers, and to discuss possible challenges in delivery of the intervention. This will enhance the fidelity of the intervention by ensuring that the intervention is implemented as intended.

### Evaluation of the intervention & data collection

#### Quantitative assessment of outcomes

The quantitative data will be collected to capture the impact of the intervention implemented by SCPs on the outcome measures caregiver burden [30], depressive symptoms [31], quality of life [32] and caregiving competence [33] of family caregivers.

Background information of the family caregivers such as age, sex, social network, relationship with the PWD, living arrangement will be included. Outcomes will be measured in both the intervention and wait list control groups at baseline prior to delivery of intervention (T1), after completion of the 10-week intervention in the intervention group (T2), and after completion of the 10-week intervention in the wait list control group (T3).

#### Qualitative assessment of stakeholders’ experiences and insights

In-depth interviews will be conducted with the two groups of key stakeholders of the project for three-fold purpose: (i) family caregivers to get an understanding of their experiences of receiving support from the SCPs through the mobile app including areas of improvement; (ii) SCPs to evaluate delivery of the intervention via the mobile app in terms of use and practicality of the app, and particularly in combination to their regular responsibilities; and (iii) Acceptability by SCPs and family caregivers in providing and receiving support through a mobile application. The interviews will be undertaken using a semi-structured interview guide and take place shortly after the 10-week intervention cycle. Even chat data of communication between the family caregivers and SCPs will be qualitatively analyzed to identify themes meaningful for future caregiving to family caregivers of minority ethnic groups by professionals.

A data monitoring committee will not be needed, as the included participants (family caregivers), are at a low risk of unintended harm.

#### Assessment of logged data

Usage of the app will be assessed by analytics information, logged on backend system, on the features of the mobile application described above.

The mHealth evidence reporting and assessment (mERA) checklist [25] will be followed to report on proposed intervention using mobile devices. The checklist of 16 items includes information on, for example, population level infrastructure, technology platform, intervention content, content testing, user feedback, fidelity of the intervention, etc.

The usability of the mobile app, through which the intervention is delivered, will be assessed by the following parameters:

1) *Usage* of the mobile app as indicated below (Derived from app data):

- Frequency of communication between family caregivers and the SCP through chat.
- Use of collection of weblinks of relevant services.
- Frequency of use of peer support through contact between fellow family caregivers.
- Use of mindfulness exercises.

2) User Experience in terms of *satisfaction* level of the interface of the mobile app and the content of the intervention (Interviews with family caregivers and SCPs).

3) User Experience in terms of *ease* of usage of the mobile application (Interviews with family caregivers and SCPs).

### Data analysis

#### Outcome analysis

The outcome analysis of the intervention will include quantitative assessment of family caregivers’ caregiving competence, reduction in caregiver burden and depression, and improvement in quality of life.

The *quantitative* data will be analyzed to describe and compare differences in caregiver competence, caregiver burden, depressive symptoms and quality of life amongst family caregivers between the baseline and post-intervention phases. Impact of the intervention on the odds of improving the specified outcomes will be assessed by regression analyses. All data will be analyzed using Statistical Package for Social Scientists. As neither blinding of participants, SCP’s or researchers are possible, pseudo anonymized data will be sent to a data analyst who will initiate the analysis or data.

The *logged* data on the mobile application will be quantitatively analyzed for associations between use of the different features of mobile app and the outcome measures.

#### Experience of intervention

The qualitative assessment of the stakeholder’s experience and insights on the intervention will be based on transcripts from in-depth interviews with family caregivers and SCPs after intervention. The qualitative data will be analyzed to explore SCPs’ experiences in terms of ease of use and practicality of the mobile app as well as its integration in the standard support that they provide. Family caregivers’ experience and insights about the use of the mobile app will be analyzed. Acceptability, by SCPs and family caregivers, of the mobile app as a medium through which to provide and receive support will also be investigated.

The contents of the chat discussions on the *logged data* of the app will be qualitatively analyzed to identify themes meaningful for future support by professionals, particularly to family caregivers of immigrant background.

## Discussion

The current study emanates from the experience of a previous intervention study which focused on providing support to family caregivers to people with dementia living at home in Sweden [34]. One of the intervention components in the study included direct support to family caregivers by social care professionals based at the municipalities delivered through a mobile application. The content of the support by the professionals was not structured and depended on needs expressed by the family caregivers themselves. During the course of this study, it was noted that family caregivers of non-European immigrant backgrounds were not availing themselves of the support services which they were entitled and had access to. Consequently, the current study shifted focus to this specific population group. It is important to add that when designing support initiatives all caregivers should be considered despite ethnic and cultural backgrounds to promote equity. The outcomes of this study aim to contribute valuable insights that can be integrated into mainstream support services benefiting not only families caring for persons with dementia but also persons with other long term non-communicable diseases.

In a recent systematic review, learning skills to manage and take care of persons with dementia was identified as one of the major needs by family caregivers [35]. Hence, the content of the direct support to the family caregivers in the current study is inspired by the iSupport manual developed by the World Health Organization [36]. The contents, in the form of a booklet, aims to equip the family caregivers with structured information on understanding dementia as a condition and its management at home, including self-care guidance designed specifically for family caregivers themselves. This booklet was developed by some of the co-authors (ZNK, MT & ÅC) and used in telephone-delivered intervention study targeted for family caregivers to persons with dementia in Malaysia. An RCT parallel to the one described in this study protocol will be conducted in India using the same booklet however adapted to the Indian context. These multiple intervention studies will provide the evidence base on the effectiveness of using digital technologies to deliver support to those who are not reached by the traditional healthcare system, yet who are crucial partners in the care of persons with progressive illnesses.

Potential limitations of the research described in this study protocol include the broad definition of the study population and the inclusion criteria of knowledge of Swedish. The study includes immigrant of non-European background who are not a homogenous group which may impact the findings of the study. The inclusion criteria of knowledge of Swedish language may lead to selection bias by leaving out participants for whom it may be even more difficult than others to understand and access health and social care services offered in Sweden. In such cases, younger generations within the family can assist the family caregivers in communicating with the social care professionals through the mobile app. There may even be availability bias as participants who take part in the research may do so as they have the time to participate in the research in addition to the time they use for their caregiving tasks, compared to others who do not participate. However, the intervention tool in the project offers the flexibility of using it at any time of day that is of convenience for the family caregivers. We even expect gender bias among the family caregivers in the study reflecting the reality of mostly women providing caregiving to PWD in the family [37, 38]. Addressing this bias in a compensatory manner is therefore challenging, but it is crucial to keep this in mind. Caregiving dynamics are evolving, impacting both motivations and approaches to caring for older individuals. Women often bear a more significant burden of care within families. There may be a lack of culturally sensitive formal services that adequately address the care needs of aging immigrants and their family caregivers. Creating an inclusive care environment that considers diverse needs is crucial, especially for older immigrants and their dedicated family caregivers [38].

Findings of the research will be published in peer-reviewed international journals irrespective of the significance level of the results. Presentations in national and international conferences, seminars/webinars and workshops, and other professional and community fora will be made to reach the general audience. If potential lessons can be taken from the intervention on social services for older persons and primary health care delivery, they will be disseminated to the relevant policy makers in the form of policy notes. Communication about the intervention project will also be done on social media and professional networks, e.g., X (formerly Twitter), Facebook, ResearchGate, LinkedIn, etc.

### Electronic supplementary material

Below is the link to the electronic supplementary material.

Supplementary Material 1

## Data Availability

Data sharing is not applicable to this article as no dataset was generated or analyzed in writing this study protocol.
