# Supplementary material for: mHealth based intervention by social care professionals to support family caregivers to persons with dementia living at home in Sweden (Caregiver Connect): a randomized controlled trial
Source: BMC Geriatr. 2024 Jun 14;24:519. doi: 10.1186/s12877-024-05106-x (PMC11177475; doi:10.1186/s12877-024-05106-x)
Supplement: Supplementary file 1 — Supplementary Material 1 [file 12877_2024_5106_MOESM1_ESM.doc]

Figure 1. SPIRIT flow chart of the schedule of enrolment, intervention, and assessments.

|  | **STUDY PERIOD** | | | | | |
| --- | --- | --- | --- | --- | --- | --- |
|  | **Enrolment** | **Allocation** | **Post-allocation** | | | **Close-out** |
| **TIMEPOINT**** | ***-t_1_*** | **0** | ***t_1_*** | ***t_2_***  ***_10 weeks_*** | ***t_3_***  ***_20 weeks_*** | ***t_x_***  ***_1 week after intervention_*** |
| **ENROLMENT:** |  |  |  |  |  |  |
| **Eligibility screen** | X |  |  |  |  |  |
| **Informed consent** | X |  |  |  |  |  |
| **Allocation** |  | X |  |  |  |  |
| **INTERVENTIONS:** |  |  |  |  |  |  |
| ***Intervention group*** |  |  |  |  |  |  |
| ***Wait list control group*** |  |  |  |  |  |  |
| **ASSESSMENTS:** |  |  |  |  |  |  |
| ***Baseline variables: Age, sex, social network, relationship with the person with dementia, living arrangement.*** | X |  |  |  |  |  |
| ***Outcome variables: Caregiver burden, depressive symptoms, quality of life, caregiving competence.*** |  |  | X | X | X | X |
| ***App analytics*** |  |  |  |  |  | X |
| ***Qualitative Interviews on satisfaction with the intervention and ease of use of the intervention tool*** |  |  |  |  |  | X |

Source: [Chan A-W, Tetzlaff JM, Altman DG, Laupacis A, Gøtzsche PC, Krleža-Jerić K, Hróbjartsson A, Mann H, Dickersin K, Berlin J, Doré C, Parulekar W, Summerskill W, Groves T, Schulz K, Sox H, Rockhold FW, Rennie D, Moher D. SPIRIT 2013 Statement: Defining standard protocol items for clinical trials. *Ann Intern Med* 2013;158:200-207.](http://www.annals.org/article.aspx?doi=10.7326/0003-4819-158-3-201302050-00583)
